# IDH2 regulates macrophage polarization and tumorigenesis by modulating mitochondrial metabolism in macrophages

**DOI:** 10.1186/s10020-024-00911-x

**Published:** 2024-09-10

**Authors:** Sung Woo Lee, Soyoon Kim, Bokyung Kim, Jung Bae Seong, Young-Ho Park, Hong Jun Lee, Dong Kyu Choi, Eunbyul Yeom, Dong-Seok Lee

**Affiliations:** 1 School of Life Sciences, BK21 FOUR KNU Creative BioResearch Group, Kyungpook National University, Daegu, 41566 Republic of Korea; 2 School of Life Sciences & Biotechnology, College of Natural Sciences, Kyungpook National University, Daegu, 41566 Republic of Korea; 3 Illimis Therapeutics Inc., Seoul, 06376 Republic of Korea; 4 National Primate Research Center, Korea Research Institute of Bioscience and Biotechnology (KRIBB), Cheongju, 28116 Republic of Korea; 5 Futuristic Animal Resource & Research Center (FARRC), Korea Research Institute of Bioscience and Biotechnology (KRIBB), Cheongju, 28116 Republic of Korea; 6 College of Medicine and Medical Research Institute, Chungbuk National University, Cheongju, Chungbuk Republic of Korea; 7 Research Institute, huMetaCELL Inc., 220 Bugwang-ro, Bucheon-si, Gyeonggi-do Republic of Korea

**Keywords:** Isocitrate dehydrogenase 2, Mitochondria, Cancer, Tumor microenvironment, Macrophage polarization

## Abstract

**Background:**

Targeting the tumor microenvironment represents an emerging therapeutic strategy for cancer. Macrophages are an essential part of the tumor microenvironment. Macrophage polarization is modulated by mitochondrial metabolism, including oxidative phosphorylation (OXPHOS), the tricarboxylic acid (TCA) cycle, and reactive oxygen species content. Isocitrate dehydrogenase 2 (IDH2), an enzyme involved in the TCA cycle, reportedly promotes cancer progression. However, the mechanisms through which IDH2 influences macrophage polarization and modulates tumor growth remain unknown.

**Methods:**

In this study, IDH2-deficient knockout (KO) mice and primary cultured bone marrow-derived macrophages (BMDMs) were used. Both in vivo subcutaneous tumor experiments and in vitro co-culture experiments were performed, and samples were collected for analysis. Western blotting, RNA quantitative analysis, immunohistochemistry, and flow cytometry were employed to confirm changes in mitochondrial function and the resulting polarization of macrophages exposed to the tumor microenvironment. To analyze the effect on tumor cells, subcutaneous tumor size was measured, and growth and metastasis markers were identified.

**Results:**

IDH2-deficient macrophages co-cultured with cancer cells were found to possess increased mitochondrial dysfunction and fission than wild-type BMDM. Additionally, the levels of M2-associated markers decreased, whereas M1-associated factor levels increased in IDH2-deficient macrophages. IDH2-deficient macrophages were predominantly M1. Tumor sizes in the IDH2-deficient mouse group were significantly smaller than in the wild-type mouse group. IDH2 deficiency in macrophages was associated with inhibited tumor growth and epithelial–mesenchymal transition.

**Conclusions:**

Our findings suggest that IDH2 deficiency inhibits M2 macrophage polarization and suppresses tumorigenesis. This study underlines the potential contribution of IDH2 expression in macrophages and tumor microenvironment remodeling, which could be useful in clinical cancer research.

**Supplementary Information:**

The online version contains supplementary material available at 10.1186/s10020-024-00911-x.

## Introduction

Apart from cancer cells, the tumor microenvironment also contains endothelial cells, fibroblasts, and immune cells. During tumor progression, fibroblasts and immune and vascular cells are actively recruited to primary tumor sites to reproduce and secrete cytokines and chemokines (Alkasalias et al. 2018). Circulating blood monocytes are also recruited to the tumor matrix (Winkler et al. 2020), where tumor cell-secreted macrophage colony-stimulating factor (M-CSF) drive their differentiation into macrophages (Laoui et al. 2014; Yi et al. 2024). Monocytes are polarized by the tumor microenvironment and differentiate into tumor-associated macrophages (TAMs), acquiring immune suppression and protumor properties (Richards et al. 2013). The accumulation in tumors of TAMs changes the tumor microenvironment, promoting tumor development and progression (Yuan et al. 2016). Significantly, the tumor microenvironment impacts the outcomes of therapeutic interventions, as shown in several previous research trials (Margolin et al. 2011; Bruchard et al. 2013; Ham et al. 2019). Hence, regulating the tumor microenvironment represents a promising strategy for inhibiting tumor progression and metastasis.

Macrophages are phagocytic cells involved in tissue homeostasis, defense mechanisms, and wound healing. Activated macrophages are classified into two phenotypes, M1 and M2, based on the expression of surface receptors, secretory factors, and functions (Boutilier and Elsawa 2021). M1 macrophages fight invading pathogenic bacteria, produce nitric oxide (NO), and exhibit inflammatory phenotypes (Wang et al. 2014). In contrast, M2 macrophages exhibit anti-inflammatory responses. Moreover, M2 macrophages provide a favorable tumor microenvironment for growth, survival, and angiogenesis (Hao et al. 2012, Sica and Mantovani 2012). TAMs typically switch to an M2-like phenotype during late-stage tumor progression and exhibit protumor activities (Pollard 2004, Sica and Mantovani 2012). Although the phenotype and function of TAMs have been well-documented, the precise mechanism underlying the regulation of macrophage polarization for inhibiting tumor growth remains unknown.

Mitochondria produce energy and metabolites necessary for the cell cycle and apoptosis. During cellular respiration, the reactants of the electron transport chain (ETC) in mitochondria transfer electrons via redox reactions. The ETC marks the culmination of glucose metabolism, which occurs via glycolysis and tricarboxylic acid (TCA) cycle. Alterations in the TCA cycle are closely related to macrophage polarization, and M1 polarization represents an impaired TCA cycle characterized by citrate accumulation (O'Neill 2015). Furthermore, the low α-ketoglutarate/succinate ratio enhances M1 macrophage activation (Angajala et al. 2018). Isocitrate is converted to α-ketoglutarate by isocitrate dehydrogenase (IDH). In addition, a study using IDH2-deficient mice revealed that IDH2 is related to tumor progression (Kim et al. 2014). However, the mechanism through which macrophages influence tumorigenesis in IDH2-deficient mice has not yet been explored sufficiently.

Here, we revealed that IDH2 deficiency in macrophages affected their polarization, inhibiting the M2-like phenotype. Moreover, IDH2 deficiency suppressed OXPHOS and induced mitochondrial fragmentation in macrophages. Furthermore, IDH2-deficient macrophages failed to promote cancer cell growth and epithelial–mesenchymal transition (EMT) in vitro and in vivo. Thus, we propose that IDH2 deficiency in macrophages is a pivotal regulator of cancer progression.

## Materials and methods

### Reagents

Murine macrophage colony-stimulating factor (mM-CSF) was obtained from PeproTech (Cranbury, NJ, USA).

### Cell culture and co-culture

LLC1 (murine lung carcinoma) cells were obtained from the American Type Culture Collection (Manassas, VA, USA). Cells were maintained in Dulbecco’s Modified Eagle’s medium (DMEM) (Welgene, Daegu, Korea) supplemented with 10% fetal bovine serum (FBS; Thermo Fisher Scientific, Waltham, MA, USA), 100 U/mL penicillin, and 100 μg/mL streptomycin (Welgene) at 37 °C. Cancer cells and bone marrow-derived macrophages were co-cultured at a ratio of 1:10 for 72 h with separation by 0.4 μm pore transwell inserts.

### Animal care and experiments

We used 8-week-old male C57BL/6 J wild-type (WT) and *IDH2*-/- mice (Kim et al. 2014). The *IDH2*-/- mice were kindly provided by Prof. J.W. Park. Mice were housed at 23 °C in a 12:12 h light/dark cycle. All animal experiments were performed according to national ethical guidelines and were approved by the Institutional Animal Care and Use Committee (IACUC) at the Kyungpook National University. These *IDH2*-/- mice were identified by PCR using primers 5′- ACTGTTCTGGAACATGCTGCC-3′ and 5′-TCCTCAAAGCATCAGGTACCG-3′ (Fig. S1). LLC1 cells (5 × 10^5^ cells in 50 μL PBS) were subcutaneously inoculated into the left flank of 8-week-old WT or *IDH2*-/- C57BL/6 J mice. Tumor volume was measured every 3 d for 15 d. Tumor sizes were calculated using the formula: (mm^3^) = (L × W2) × 0.5. The Student’s *t*-test (two-tailed) was used to compare the differences in the results of the two groups. The experiment was terminated when tumor burden limits were not exceeded (< 1 cm^3^) in compliance with IACUC and Animal Cancer Research guidelines. After the experiment ended, mice were euthanized, and tumor masses were subjected to analysis. No mice died prior to reaching the endpoint of the study.

### Primary culture of bone marrow-derived macrophages (BMDMs)

Preparation of BMDMs were based on the protocol described (Weischenfeldt and Porse 2008; Zhang et al. 2008; Trouplin et al. 2013). Briefly, Femurs were isolated from 8 to 12 weeks old WT and *IDH2-/-* mice. The distal and proximal ends of the femur were cut using sterile scissors, and the bone marrow (BM) cavity was flushed with DMEM to collect BM cells. BM cells incubated in DMEM supplemented with 10% FBS, antibiotics, and 10 ng/mL mM-CSF for 7 days to generate BMDMs. On day 3, removed culture media with unattached cell and replaced to fresh media. The BMDMs were gently resuspended in freezing media and aliquoted into individual cryovials. The vials were initially stored at − 80 °C for 24 h, followed by transfer to a liquid nitrogen tank for cryopreservation for additional experiments.

### Western blot analysis

Total protein isolate was extracted using the PRO-PREP protein extraction solution (iNtRON Biotechnology, Seongnam, Korea) and quantified using an Infinite F50 microplate reader (Tecan, Männedorf, Switzerland). Samples containing equal amounts of protein (20 μg) were resolved by performing SDS-PAGE using 8–15% gradient gels and then transferred onto nitrocellulose membranes (Pall Corporation, Pensacola, FL, USA). Membranes were incubated at 4 °C with primary antibodies against iNOS, STAT3, p-STAT3, AMPK, p-AMPK (Cell Signaling Technology, Danvers, MA, USA), fibronectin, N-cadherin, GAPDH (Santa Cruz Biotechnology Inc., Dallas, TX, USA), vimentin (Abfrontier, Seoul, Korea), CD206 (Invitrogen, USA), Ki67 (Sigma-Aldrich, Saint Louis, MO, USA), and Arg1 (BD Biosciences, NJ, USA). Membranes were washed three times with 10 mM Tris–HCl (pH 7.5) containing 150 mM NaCl and 0.1% Tween-20 (TBST) and then incubated overnight at 4 °C with horseradish peroxidase-conjugated goat anti-rabbit and anti-mouse secondary antibodies (Thermo Fisher Scientific). Membranes were washed six times with TBST to remove non-specifically bound secondary antibodies. Immunoreactive bands were detected using the Clarity Western ECL Substrate (Bio-Rad, CA, USA) according to the manufacturer's instructions, and band intensities were analyzed using the Multi Gauge version 3.0 software (Fujifilm, Japan).

### Real-time quantitative PCR (RT-qPCR) assay

Total RNA was extracted from tumor tissues and cells using the Ribospin™ II kit (GeneAll, Seoul, Korea). RNA was converted to cDNA using the Primer Script RT Reagent kit (TaKaRa, Japan). PCR reactions were assembled in a 96-well plate (Thermo Fisher Scientific) using the TB green master mix (TaKaRa). The list of used primers is provided in Supplementary Table 1.

### Immunohistochemistry (IHC)

Tumor tissues collected from each animal group were fixed in 4% paraformaldehyde, embedded using a Tissue-Tek O.C.T. compound (Sakura Finetek, USA), cut into sections of 10 μm thickness, and subjected to immunohistochemistry. Briefly, tissue sections were first incubated with a blocking solution (general chlorine serum 10%; Gibco, New Zealand) followed by incubation with anti-CD206 (Invitrogen), anti-iNOS (Cell Signaling Technology), and anti-Ki67 (Sigma-Aldrich) antibodies at 4 °C. Subsequently, tissue sections were labeled using Alexa Fluor 594 binding and Alexa Fluor 488 secondary antibodies (Molecular Probes, OR, USA) for 1 h and observed under an LSM-800 confocal microscope.

### Immunocytochemistry (ICC)

Cells were seeded on 24 mm circular glass coverslips (Paul Marienfeld, Lauda-Königshofen, Germany) coated with 0.1% poly D-lysine, co-cultured with cancer cells for 72 h, washed with PBS, and fixed with 4% paraformaldehyde for 1 h. Then, the cells were washed three times with PBS and incubated with primary antibodies against CD206 (Thermo Fisher Scientific), iNOS (Cell Signaling Technology), and Ki67 (Cell Signaling Technology). Subsequently, cells were labeled with Alexa Fluor 594 and Alexa Fluor 488 secondary antibodies (Thermo Fisher Scientific) for 1 h. Each cover slip was then mounted on a slide using VECTOR SHIELD (VECTOR LABORATORIES, CA, USA).

### Flow cytometry

BMDMs were co-cultured with LLC1 cells for 72 h or treated with 10 ng/mL IL-4 for 24 h. BMDMs were gently detached using a cell scraper. Next, 1 × 10^6^ BMDM cells were stained with 100 μL of antibodies in cell staining buffer (CD11b-phycoerythrin (PE)-conjugated antibody (Thermo Fisher Scientific), CD86-PerCP-Cyanine5.5 (PerCP-Cy5.5) conjugated antibody (Biolegend; San Diego, CA, USA)), or CD206-fluorescein fluorescein isothiocyanate-(FITC) conjugated antibody (Biolegend)). Samples were analyzed using a FACS Verse™ (BD Biosciences).

### Alpha-ketoglutarate (α-KG) assay

After 72 h of culturing, cells were washed with PBS and resuspended in 500 μL ice-cold α-KG Assay Buffer (ab83431, Abcam; Cambridge, UK) according to the manufacturer’s instructions. Cells were quickly homogenized by pipetting up and down a few times and then centrifuged to remove any insoluble material. Subsequently, ice-cold PCA was added to the homogenate (final concentration: 1 M), and the mixture was vortexed briefly to mix well. The sample was centrifuged, and the supernatant was transferred to a fresh tube. Excess PCA was precipitated by adding ice-cold 0.2 M KOH and brief vortexing. After neutralizing the sample to ensure the pH was 6.5–8, it was centrifuged at 13,000 rpm and 4 °C for 15 min, and the supernatant was collected. Next, 50 μL α-KG standard or cell sample was added to each well of 96-well plates. Next, the reaction mix was added to each well and incubated in the dark at 37 °C for 30 min. Finally, the absorbance was measured at 570 nm using a microplate reader.

### Metabolic flux assays (Seahorse assay)

BMDMs were co-cultured with LLC1 cells or cultured alone for 72 h. BMDM from different conditions were gently detached with cell scraper and resuspended at the same concentration. 2 × 10^4^ cells were seeded in each well of an assay plate one day prior to the experiment. Before the assay, the plate was incubated at 37 °C in a non-CO2 incubator for one hour. Following this initial incubation, XF Running Media was dispensed into each well. Oxygen consumption rate (OCR) and extracellular acidification rate (ECAR) were measured using an XF96 Seahorse Extracellular Flux Analyzer, following the manufacturer’s instructions. The basal OCR was measured, followed by continuous assays using serial injections of 2.5 µM Oligomycin, 1 µM Carbonyl cyanide 4-(trifluoromethoxy) phenylhydrazone (FCCP), and a combination of 1 µM Rotenone and 1 µM Antimycin A. For the measurement of glycolytic function, cells were subsequently treated with 10 mM glucose, 2.5 µM Oligomycin, and 50 mM 2-Deoxyglucose (2-DG). After analysis, the plates were stored. The cell counts in each well were subsequently measured, and the values were normalized based on the number of cells per well.

### NO Detection

The amount of NO in the culture supernatant was measured using a commercially available NO detection kit (iNtRON Biotechnology) according to the manufacturer’s instructions. Absorbance was measured at 450 nm using a microplate reader.

### Mitochondrial imaging and length measurement

Briefly, 5 × 10^4^ cells were seeded on a 24 mm circular glass coverslip (Paul Marienfeld, Lauda-Könichsoffen, Germany) coated with 0.1% poly D-lysine, cultured for 72 h, and washed with PBS. This was followed by adding a prewarmed (37 °C) solution containing a MitoTracker probe (Thermo Fisher Scientific). After incubating for 45 min, cells were washed twice with PBS, fixed with 4% paraformaldehyde in PBS for 1 h, and washed three times with PBS. Finally, the coverslip was mounted on a slide using VECTASHIELD mounting media (VECTOR LABORATORY). Images were obtained using an LSM-800 confocal microscope (Carl Zeiss, Oberkochen, Germany) equipped with a planar chromaticity 100 × /1.40 oil DIC M27 target lens. Images were processed using the Zeiss LSM Image Inspector, ZEN 2009 Light Edition (Carl Zeiss). Mitochondrial lengths were measured using the ImageJ software (NIH, MD, USA), and average values were calculated using more than 50 mitochondria per cell for 20 cells. Mitochondria were divided into different categories according to their length: < 1 μm, 1–3 μm, and > 3 μm.

### Confocal laser microscopy

Images were obtained using an LSM-800 confocal microscope (Carl Zeiss) equipped with a Plan- Neofluar 20 × /0.50 M27 objective lens and processed using a Zeiss LSM Image examiner and ZEN lite 2009 edition software (Carl Zeiss).

### Clonogenic assay

A total of 1 × 10^5^ LLC1 cells were seeded in the lower chamber of a transwell plate, whereas BMDMs were seeded in the upper chamber. After 72 h of culture, cells were washed twice with PBS, fixed with 4% paraformaldehyde (Sigma-Aldrich), and stained with 2% crystal violet (Sigma-Aldrich).

### Migration assay

To evaluate the migration and infiltration capability of cancer cells, we used a chamber (SPL) along with a Transwell insert (pore size: 8 μm). The insert-containing chamber was coated with 2 μg collagen type I (Sigma-Aldrich), and cancer cells were seeded. BMDMs were seeded at the bottom of the plate, and the cells were cultured at 37 °C for 72 h. Subsequently, the Transwell insert was removed from the plate, and the remaining cells were smoothly removed. Cells in the insert chamber were fixed with 4% paraformaldehyde/PBS, stained with 2% crystal violet, washed with PBS, and dried. The number of cells migrated into the Μatrigel was calculated in five representative fields (× 100) per insert.

### Magnetic-activated cell sorting (MACS)

Transplanted tumors were dissociated with collagenase (Sigma-Aldrich) and dispase II (Roche, Switzerland) using gentleMACS™ Octo Dissociator with Heaters (Miltenyi Biotec, Germany). Resuspended cells were mixed with anti-F4/80 microbeads (Miltenyi Biotec) and incubated on ice for 15 min. Cells were then washed with HBSS–BSA buffer and centrifuged at 300 × *g* and 4 °C for 10 min. The QuadroMACS™ Separator (Miltenyi Biotec) was used for isolating cells, which were then resuspended in the HBSS–BSA buffer and applied to the LS column (Miltenyi Biotec). Finally, cells were washed with HBSS–BSA buffer and collected in 15 mL tubes.

### Statistical analysis

Data are presented as the mean ± standard error of the mean (SEM) of three or more independent experiments. Statistically significant differences were determined by t-test, one-way ANOVA, and two-way ANOVA using the GraphPad Prism 8 software (San Diego, CA, USA): One-way ANOVA and two-way ANOVA using Tukey’s post-hoc test for intergroup comparisons. Results with p < 0.05 were considered statistically significant. Two and three asterisks indicate p-values of < 0.01 and < 0.001, respectively.

## Results

### IDH2 deficiency reduces mitochondrial function

**Fig. 1:**
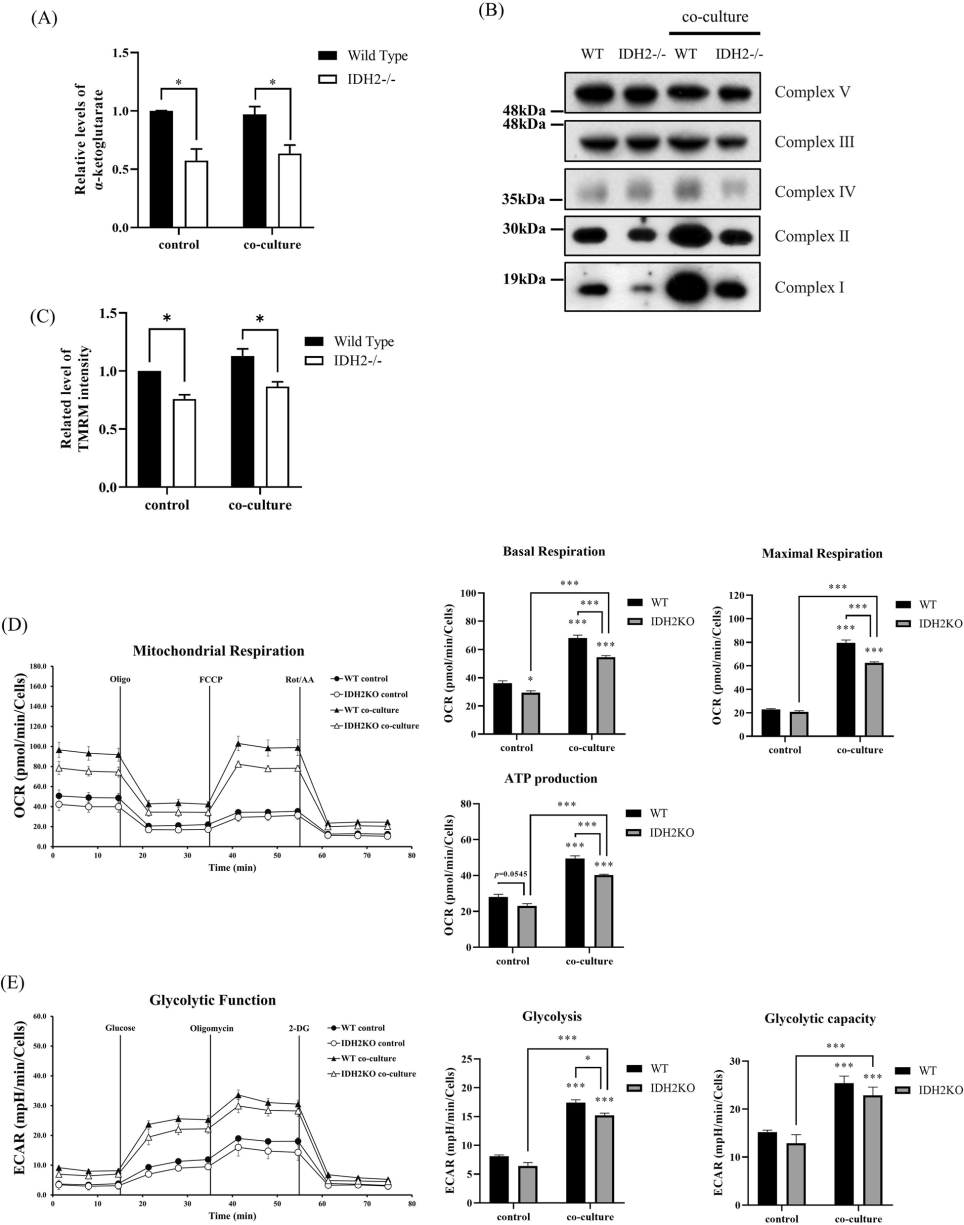
IDH2 deficiency disrupts mitochondrial metabolic function in macrophages. WT or IDH2-deficient BMDMs were cultured alone or co-cultured with LLC1 cells for 72 h. **A** Expression level of α-ketoglutarate in BMDMs co-cultured with cancer cells. **B** Western blot analysis reflecting OXPHOS status in BMDMs co-cultured with cancer cells. **C** Flow cytometry analysis of mitochondrial membrane potential in BMDMs co-cultured with cancer cells. **D** and **E** Seahorse analysis results for OCR and ECAR of BMDMs from baseline condition or co-cultured with LLC1. **D** basal respiration, Maximal respiration, ATP production. **E** Glycolysis, Glycolytic capacity. Data are presented as the mean ± SEM of at least three independent experiments (*p < 0.05, **p < 0.01, ***p < 0.001, two-way ANOVA with Tukey’s post hoc test)

The TCA cycle supplies NADH and FADH2, which act as the electron carriers in the ETC (Nunnari and Suomalainen 2012). IDH2 converts isocitrate to α-ketoglutarate and is involved in the TCA cycle. We first analyzed the level of α-ketoglutarate in WT and IDH2-knockout macrophages, both with and without LLC1 co-culture. The level of α-ketoglutarate decreased in IDH2-deficient macrophages (Fig. 1A). Next, we investigated whether OXPHOS was affected in macrophages co-cultured with cancer cells. The OXPHOS system consists of five complexes and create the proton motif force of the inner mitochondrial membrane (IMM) through NADH and FADH2 supplied through the TCA cycle (Tang et al. 2020). Compared with WT macrophages, OXPHOS complex I and II were suppressed in IDH2-deficient macrophages regardless of co-culturing with cancer cells. Additionally, complexes III and IV showed a tendency to decrease when co-cultured with cancer cells. (Fig. 1B). Mitochondrial membrane potential (Δψ) is a metabolic marker that drives the generation of ATP by mitochondria (Zorova et al. 2018). In this study, the IDH2-deficient macrophage had a lower mitochondrial membrane potential compared to WT cells in the TMRM assay (Fig. 1C). Then, regardless of co-culture with cancer cells, the mitochondrial membrane potential reduced in IDH2-/- macrophages. Additionally, we evaluated OCR and ECAR using the Seahorse XFe96 extracellular flux analyzer to assess mitochondrial function. Compared to WT macrophages, basal respiration was slightly reduced in the control group, but no significant changes were observed in ATP production. However, after co-culture with LLC1 cells, the OCR of macrophages was significantly increased. Furthermore, a decreased OCR was measured in IDH2-deficient macrophages compared to WT macrophage (Fig. 1D). ECAR also increased significantly by co-culture with LLC1, but depending on the expression of IDH2 was not significant (Fig. 1E). These results indicated that IDH2 deficiency reduced mitochondrial function in macrophages co-cultured with/without cancer cells.

### IDH2 deficiency induced morphological changes in macrophage mitochondria

**Fig. 2:**
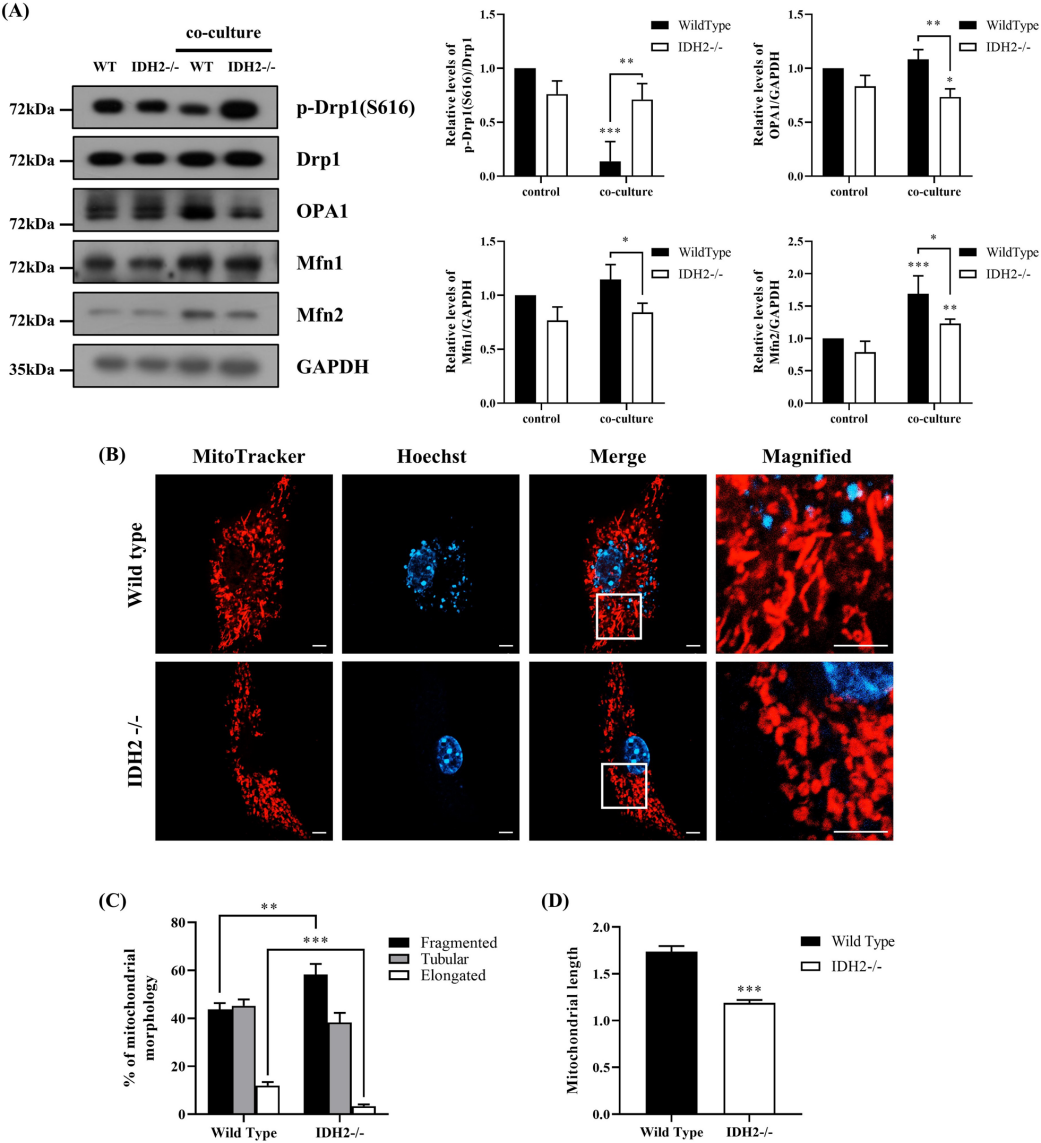
Mitochondrial fission increased in IDH2-deficient macrophages co-cultured with cancer cells. **A** Western blot analysis of p-Drp1, Drp1, OPA1, Mfn1, and Mfn2 expressions in BMDMs cultured alone or co-cultured with cancer cells. **B** Wild-type and IDH2-deficient BMDMs were co-cultured with LLC1 lung carcinoma cells. Mitochondrial morphology in BMDMs was observed by confocal microscopy following staining with MitoTracker and Hoechst. **C, D** Mitochondria were classified according to length, ranging between < 1 μm, 1–3 μm, and > 3 μm. The mitochondrial length was measured using ImageJ software. Data are presented as the mean ± SEM of at least three independent experiments (*p < 0.05, **p < 0.01, ***p < 0.001, *t*-test and two-way ANOVA with Tukey’s post hoc test)

Polarized macrophages exhibit metabolic characteristics in vitro, which are closely linked to mitochondrial function (Jha et al. 2015, Williams and O'Neill 2018). Another feature is that the type-polarized macrophages possess mitochondria of different morphological features. For instance, the mitochondria of M1 macrophages are much shorter than M2 macrophages (Li et al. 2020). Therefore, we first investigated mitochondrial dynamics in macrophages co-cultured with cancer cells using Western blot analysis. Phosphorylation of Drp1, which is the regulator of mitochondrial fission, at serine 616 leads to its activation and recruitment by mitochondria (Youle and van der Bliek 2012). Initially, expression of most mitochondrial dynamics related proteins in baseline level appeared to be very slightly lower in IDH2-dificient macrophage compared to WT. The level of p-Drp1(S616) as mitochondrial fission marker was significantly decreased in WT macrophages after co-culture with cancer cells (LLC1), but not in IDH2-deficient macrophages. Expression of mitochondrial fusion markers OPA1, MFN1, and MFN2 was significantly increased after co-culture in WT macrophages. In contrast, these fusion markers were significantly decreased in IDH2-deficient macrophages compared to WT macrophages (Fig. 2A). In addition, compared with the WT, *IDH2*-/- macrophages had fragmented mitochondria (Fig. 2B) as revealed by confocal microscope. Notably, the percentage of fragmented mitochondria was increased in IDH2-deficient macrophages, whereas that of elongated mitochondria was decreased (Fig. 2C). Furthermore, mitochondrial length was significantly reduced in *IDH2* knockout macrophages compared with in the WT (Fig. 2D). These results demonstrate that IDH2 deficiency caused morphological changes in macrophage mitochondria.

#### IDH2 deficiency alters M1/M2 polarization in *cancer* cell co-culture

**Fig. 3:**
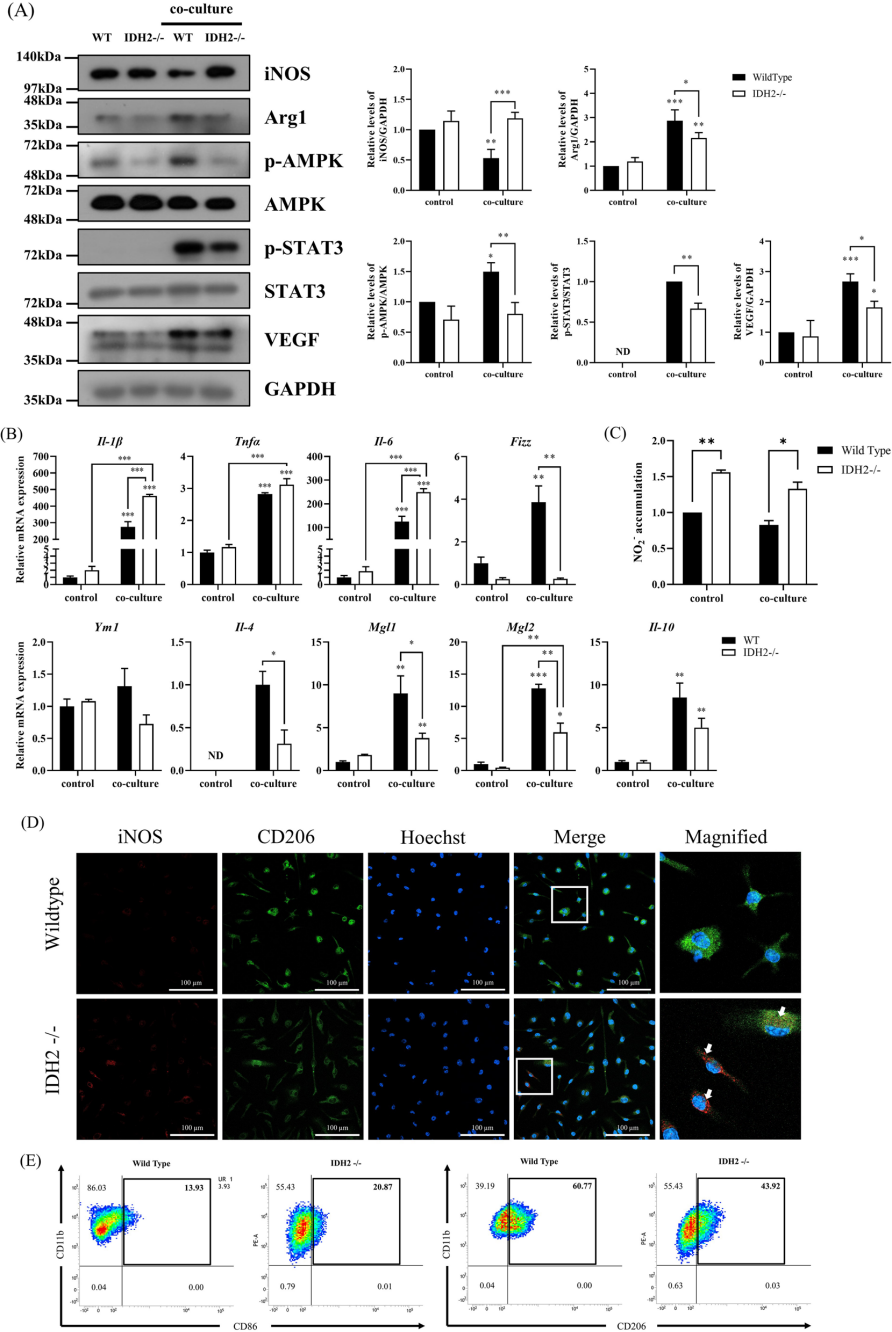
IDH2 deficiency inhibited M2 polarization in macrophages. WT or IDH2-deficient BMDMs were cultured alone or co-cultured with LLC1 cancer cells for 72 h. **A** Western blot analysis showing iNOS, p-AMPK, p-STAT3, and VEGF expressions in BMDMs co-cultured with or without cancer cells. **B** Relative levels of mRNA expressions of *Tnfα*, *Il-1β*, *Il-6*, *Fizz1*, *Il-10*, *Ym1*, *Il-4*, *Mgl1*, and *Mgl2* measured by real-time qPCR. **C** Relative NO_2_ levels in co-cultured BMDMs were measured at 450 nm using a microplate reader. **D** Immunocytochemical analysis of iNOS expression in co-cultured BMDMs. **E** Comparison of macrophage polarization to M1/M2 subtypes as a percentage of CD86 + /CD11b + or CD206 + /CD11b + macrophages using flow cytometry. ND: No data, Data are presented as the mean ± SEM of at least three independent experiments (*p < 0.05, **p < 0.01, ***p < 0.001, two-way ANOVA with Tukey’s post hoc test)

To investigate the mechanism through which IDH2 affects polarization in macrophages co-cultured with cancer cells (LLC1), we analyzed the expression of polarization-specific protein markers using Western blot analysis. After LLC1 co-culture, polarization-specific protein markers were significantly elevated, with the exception of iNOS. Compared to WT macrophages, iNOS levels, which are indicative of M1 macrophage polarization, were increased in IDH2-deficient macrophages. Conversely, Arg1 levels, a marker for M2 macrophages, were decreased in IDH2-deficient macrophages. Moreover, the extent of changes in phosphorylated AMP-activated protein kinase (pAMPK) and STAT3, known markers of M2 polarization, was reduced in IDH2-deficient macrophages after co-culture with LLC1 cells. Vascular endothelial growth factor (VEGF) contributes to tumor initiation or tumor formation via facilitating angiogenesis and affects the function of immune cells in the tumor microenvironment (Goel and Mercurio 2013). We found that the VEGF level was also decreased in IDH2-deficient macrophages. Baseline levels of p-STAT3 were too low to be quantified (Fig. 3A). Subsequently, we evaluated the level of mRNA expression of M1- and M2-related cytokines and signaling markers. As shown in Fig. 3B, most of macrophage polarization markers were increased after cancer co-culture. After co-culture, compared with WT macrophages, the expression of genes encoding M1-related markers, Il-1β, and Il-6, was significantly increased in IDH2-deficient macrophages, whereas those encoding M2-related markers, Fizz1, Il-4, MglL1, and Mgl2, was significantly decreased. The baseline expression of il-4 was too low to be defined as a value. In addition, we analyzed NO_2_^−^ generation to determine whether the inflammatory response was enhanced following IDH2 knockout. We observed increased NO_2_^−^ production in IDH2-deficient macrophages co-cultured with cancer cells (LLC1) (Fig. 3C). To evaluate M1/M2 polarization in those co-cultured with cancer cells (LLC1), we identified macrophages using confocal microscopy and flow cytometry. The level of CD206, an M2 macrophage marker, decreased in IDH2-deficient macrophages, whereas iNOS and CD86 levels, M1 macrophage markers, tended to increase (Fig. 3D and E). These findings implied that IDH2 deficiency imparts macrophages with characteristics similar to the M1 subtype and inhibits their polarization to the M2 subtype. IL-4 and IL-13 modulate macrophage phenotypes by inducing macrophage polarization to the M2 subtype (Gordon and Martinez 2010). Therefore, we analyzed whether IDH2-deficient macrophages showed reduced capability of undergoing M2 polarization under IL-4 treatment. We found that the percentage of differentiated WT M2 macrophages was almost 87.39%, whereas that of IDH2-deficient M2 macrophages was only 72.95% (Fig. S2). These results indicate that IDH2 is important in differentiating macrophages to the M2 subtype.

#### IDH2-deficient macrophages reduced *cancer* progress and metastasis

**Fig. 4:**
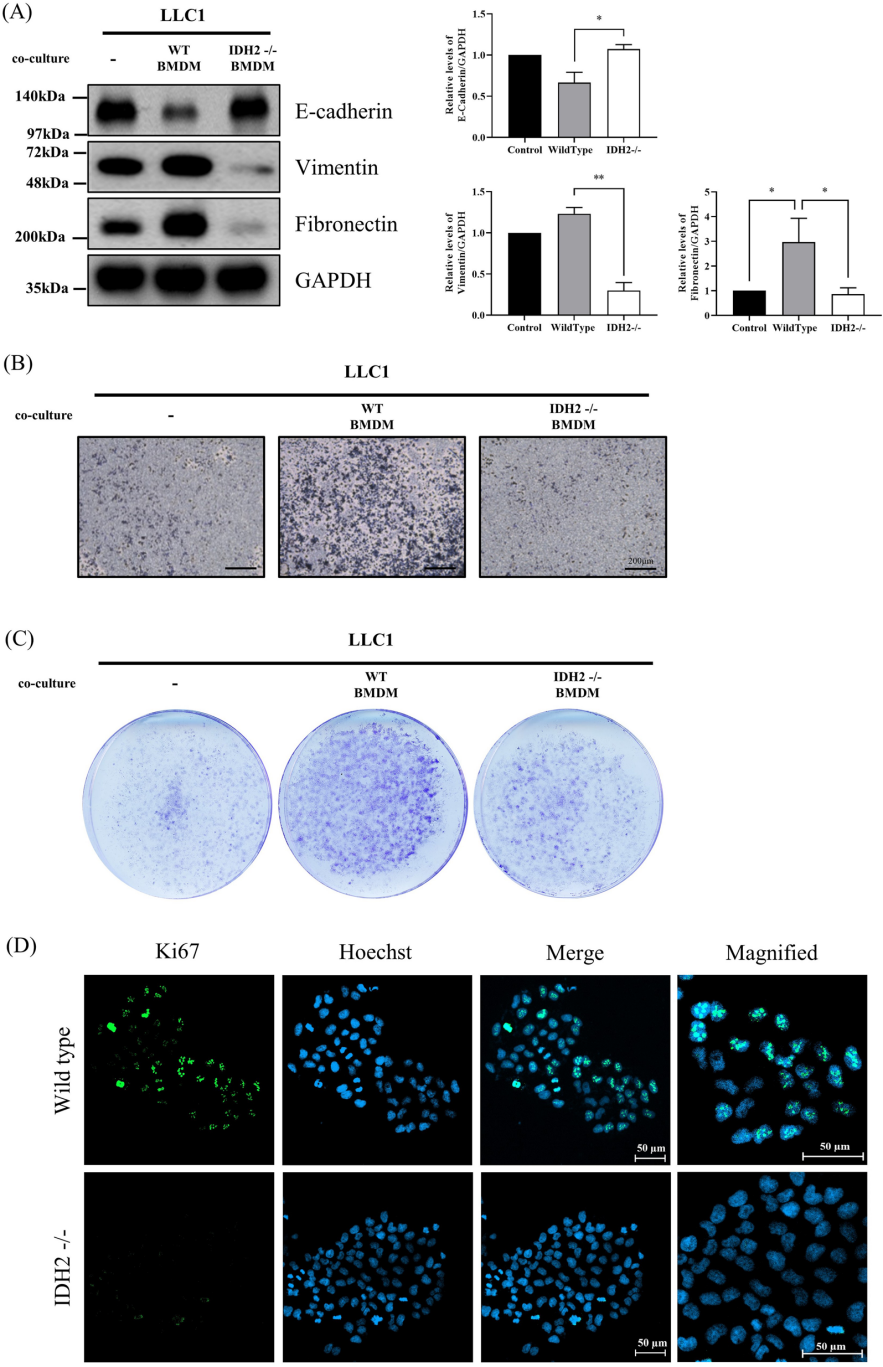
IDH2-deficient macrophages reduced cancer proliferation and metastasis. LLC1 cells were cultured alone or co-cultured with WT or IDH2-deficient BMDMs for 72 h. **A** Western blot analysis of E-cadherin, vimentin, and fibronectin expression in co-cultured LLC1 cells. **B** Clonogenic assay of LLC1 cells co-cultured with macrophages. **C** Migration assays of LLC1 cells co-cultured with macrophages. **D** Immunocytochemical analysis of staining intensity using a Ki67 antibody in co-cultured LLC1 cells. Data are presented as the mean ± SEM of at least three independent experiments (*p < 0.05, **p < 0.01, ***p < 0.001, one-way ANOVA with Tukey’s post hoc test)

EMT is a key characteristic of cancer cells with metastatic ability (Yeung and Yang 2017). Associated molecular features include altered expression of cell junction molecules, collapsing of tight junctions, and increased mesenchymal markers (Brabletz et al. 2018). We investigated the expression levels of EMT markers in cancer cells (LLC1) co-cultured with macrophages using Western blotting. In cancer cells (LLC1) co-cultured with IDH2-deficient macrophages, the expression of E-cadherin, a cell adhesion protein, remained unchanged, whereas those of mesenchymal markers vimentin and fibronectin were decreased compared with their expressions in only cancer cells (LLC1) (Fig. 4A). In contrast, co-culturing with WT macrophages upregulated the expression of vimentin and fibronectin in cancer cells, while decreasing E-cadherin expression (Fig. 4A). Based on these findings, we speculated that IDH2 deficiency in macrophages inhibits the metastatic ability of cancer. Next, we performed a co-culture using a transwell system to determine the mechanism through which IDH2-deficient macrophages influence tumor features such as clonogenicity and migration. The mobility and migratory ability of cancer cells (LLC1) were suppressed when they were co-cultured with IDH2-deficient macrophages (Fig. 4B). Moreover, we observed that colony formation was decreased in cancer cells (LLC1) co-cultured with IDH2-deficient macrophages compared to those co-cultured with WT macrophages (Fig. 4C). In addition, to confirm the proliferation capability of cancer cells, we performed ICC to evaluate Ki67 expression in cancer cells (LLC1) co-cultured with macrophages. Ki67 is a proliferation marker closely related to tumor cell proliferation and growth. We found that Ki67 expression was significantly reduced in cancer cells co-cultured with IDH2-deficient macrophages (Fig. 4D). These results suggest that IDH2 deficiency in macrophages suppresses cancer progression.

### Tumor growth was suppressed in IDH2-knockout mice

**Fig. 5:**
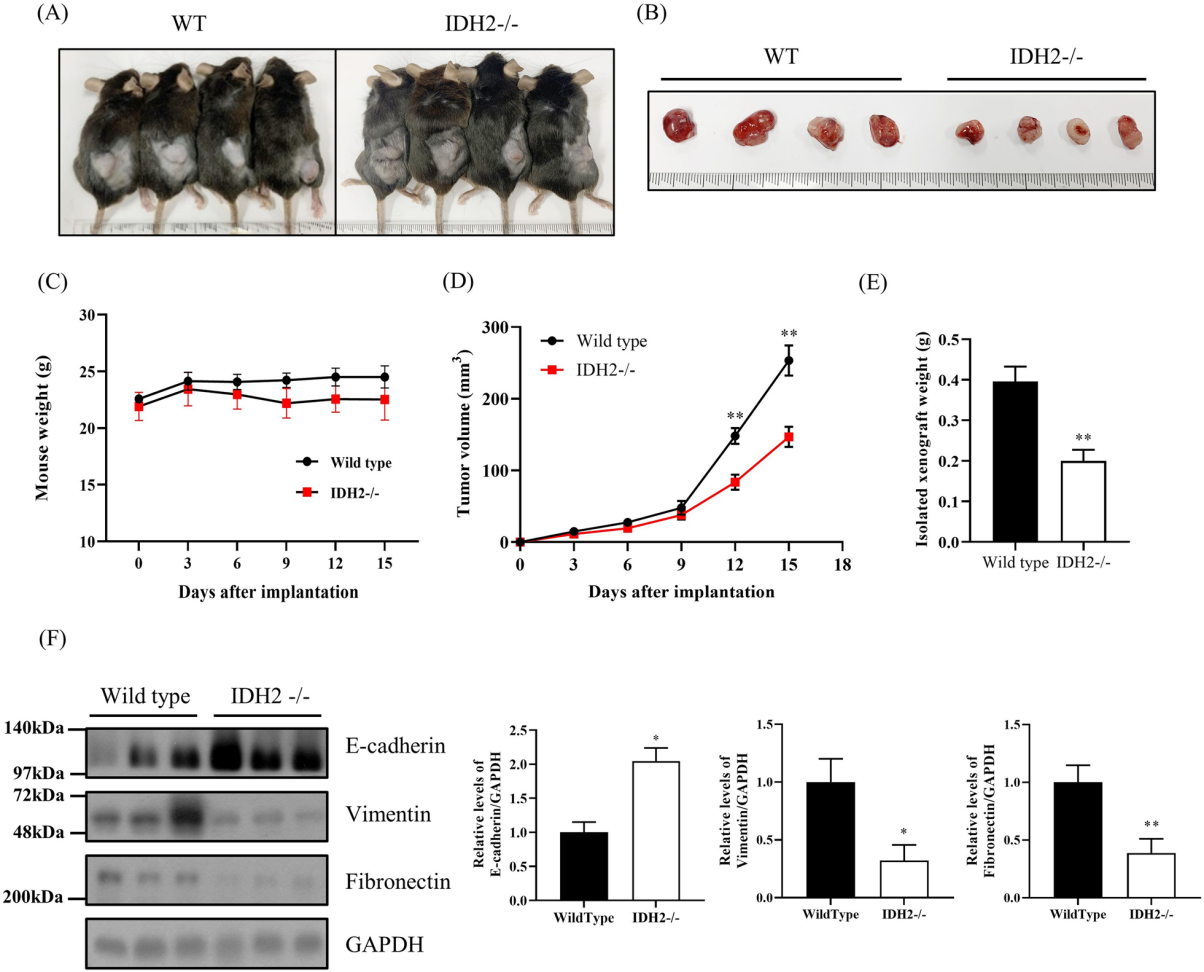
Tumor growth was suppressed in *IDH2*-knockout mice. **A** Representative image of tumor transplantation mouse model. WT and *IDH2*-/- mice were subcutaneously injected with LLC1 cancer cells into the left flank (n = 6 per group). **B** Representative image of harvested tumor masses. **C, D** Tumor growth curves for the transplantation mouse groups (WT and IDH2-/- mice). Tumor volume was calculated at 3 d intervals from the day of injection until day 15. Tumor volume was calculated using the formula: (length × width.^2^)/2. **E** Weight of the excision of the tumors. **F** Western blot analysis of E-cadherin, vimentin, and fibronectin expression in sorted macrophages in mouse tumors. Data are presented as the mean ± SEM of at least three independent experiments (*p < 0.05, **p < 0.01, ***p < 0.001, *t*-test)

We created a tumor transplantation mouse model to explore the mechanism through which IDH2 affects cancer proliferation. Thus, we subcutaneously injected cancer cells (LLC1, lung carcinoma cells) into 8-week-old WT and IDH2-deficient mice. We evaluated the size and weight of resulting tumors every 3 days and resected them 15 days after cancer cell injection. As shown in Fig. 5A and B, the average tumor size in the IDH2-deficient mouse group was significantly smaller than in the WT mouse group. Moreover, the volume and weight of tumors in *IDH2*-knockout mice were smaller than those in WT mice (Fig. 5D–E) despite no differences in body weights (Fig. 5C). We also investigated the effect of IDH2 deficiency on the EMT pathway. Similar to our in vitro experimental results, the expression of E-cadherin in IDH2-deficient mice was significantly higher in IDH2-deficient mice than in WT mice, whereas the expression of vimentin and fibronectin was lower (Fig. 5F). These results indicated that IDH2 deficiency inhibits cancer cell growth in tumor microenvironments and support the hypothesis that IDH2 could be an important factor in controlling cancer progression.

### IDH2 deficiency in macrophages inhibited M2 polarization in vivo

**Fig. 6:**
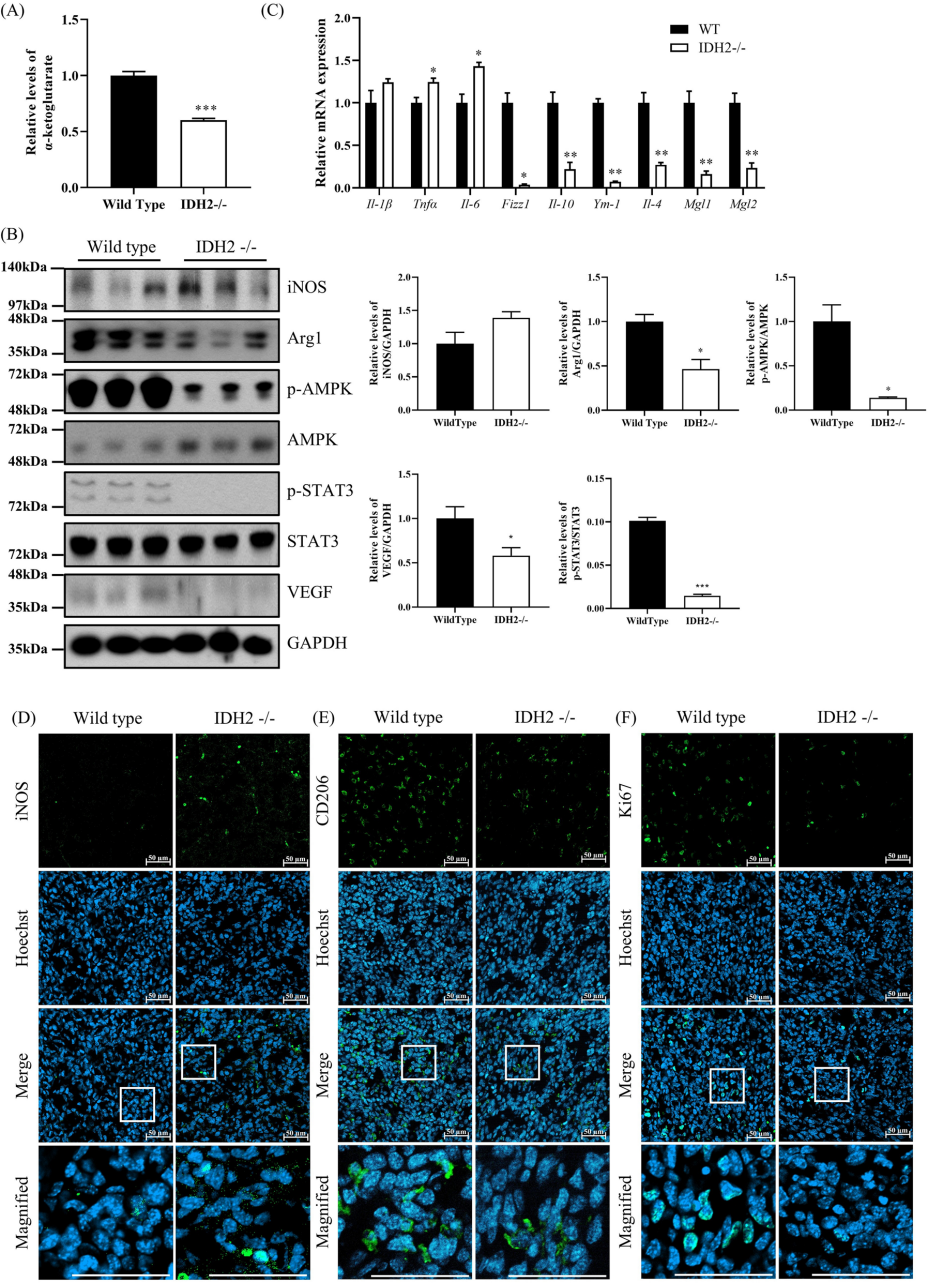
IDH2 deficiency in macrophages inhibited M2 polarization in vivo. TAMs of WT and IDH2-deficient mice isolated using MACS sorting. **A** Microplate reader analysis of α-ketoglutarate expression levels in TAMs of WT and IDH2-deficient mice. **B** Western blot analysis of iNOS, p-AMPK, p-STAT3, and VEGF expression levels in TAMs of WT and IDH2-deficient mice. **C** Relative mRNA expression levels of *Tnfα*, *Il-1β*, *Il-6*, *Fizz1*, *Il-10*, *Ym1*, *Il-4*, *Mgl1*, and *Mgl2* measured by real-time qPCR. **D, E, F** Immunohistochemical analysis of iNOS, CD206, and Ki-67 expression in mouse tumors. Data are presented as the mean ± SEM of at least three independent experiments (*p < 0.05, **p < 0.01, ***p < 0.001, *t*-test)

We isolated macrophages using MACS to investigate TAM phenotype inside tumors of WT and IDH2-deficient mice. First, we analyzed the level of α-ketoglutarate in TAMs of WT and IDH2-deficient mice. Similar to our in vitro experimental results, the level of α-ketoglutarate was decreased in IDH2-deficient TAMs (Fig. 6A). In addition, the level of iNOS, an M1 macrophage marker, was increased in TAMs of IDH2-deficient mice, whereas that of Arg1, an M2 macrophage marker, was decreased. Moreover, the p-AMPK and p-STAT3 levels and VEGF expression were also reduced in TAMs of IDH2-deficient mice, indicating that most IDH2-deficient TAMs did not differentiate into M2 macrophages and that cancer progression was inhibited in IDH2-deficient mice (Fig. 6B). Furthermore, IDH2 deficiency in macrophages reduced the mRNA levels of the M2-related markers *Fizz1*, *Il-10*, *Ym1*, *Il-4*, *Mgl1,* and *Mgl2*, while significantly increasing the expression of the M1-related markers, *Tnfα* and *Il6* (Fig. 6C). These results indicate that M2 macrophage polarization was inhibited in IDH2-deficient mice. We also performed IHC using tumor tissue sections to observe the levels of iNOS and CD206 proteins. The level of iNOS, an M1 macrophage marker, was increased in IDH2-deficient mice compared with WT mice (Fig. 6D). In contrast, the CD206 level was decreased in IDH2-deficient mice, consistent with our in vitro experimental results (Fig. 6E). Further, the number of Ki67-expressing cells was significantly reduced in tumor tissues of IDH2-deficient mice (Fig. 6F). These findings demonstrated that IDH2 deficiency inhibits M2 macrophage polarization and suppresses cancer progression.

## Discussion

There are three types of IDH: IDH1, IDH2, and IDH3. Among these, IDH2, located in mitochondria, converts isocitrate to α-ketoglutarate in the TCA cycle. Recent studies have suggested that wild-type IDH2 promotes cancer growth, whereas IDH2 deficiency suppresses tumorigenesis (Kim et al. 2014; Li et al. 2018). However, the effect of IDH2-deficient macrophages on cancer progression has not been extensively researched. In this study, for the first time, we studied the mechanisms through which IDH2 deficiency in macrophages affected tumor growth using *IDH2*-knockout mice. Our results indicated that mitochondrial dysfunction in *IDH2*-knockout macrophages was associated with the M1-like macrophage phenotype and tumorigenesis. Thus, our findings demonstrated that IDH2 is a key component of macrophage polarization in the tumor microenvironment.

Despite IDH2 knockout in macrophages, α-ketoglutarate levels decreased, maintaining at about only 50% (Figs. 1A and 6A). This could be due to the compensatory functions of IDH1 and IDH3 and the conversion of L-glutamine to α-ketoglutarate via the glutamine pathway (Yoo et al. 2020; Liu et al. 2021). In this regard, to assess the influence of IDH2 deficiency on glutamine uptake and metabolic pathways, we examined mRNA expression levels of the glutamine uptake transporter and rate-limiting enzymes in each glutamine metabolic pathway in IDH2-deficient macrophages. As shown in Figure S3, the glutamine metabolism signature genes were mostly increased in IDH2-deficient macrophages compared with WT macrophages. Therefore, we predicted that there would be changes in the glutamine metabolic pathway, which allows for the synthesis of α-ketoglutarate in the compensatory circuit for metabolic defects on *IDH2* deficiency in macrophages.

Mitochondrial dysfunction reportedly occurs during the M1 polarization (Van den Bossche et al. 2016). In contrast, highly activated OXPHOS has been related to the M2 polarization (Angajala et al. 2018). The ETC generates ATP via OXPHOS in the mitochondrial inner membrane. Meanwhile, OXPHOS caters to many important processes, including redox balance, calcium homeostasis, inflammatory signaling, and apoptosis (Nunnari, Suomalainen 2012). Recently, Liu et al*.* indicated that α-ketoglutarate inhibited the activation of IKKβ, subsequently inhibiting the NF-κB signaling pathway and reducing proinflammatory cytokine production (Liu et al. 2017). In IDH2-/- macrophages, α-ketoglutarate generation was reduced, disrupting the TCA cycle. Thus, complexes I, II, and IV in OXPHOS were suppressed, ultimately decreasing the mitochondrial membrane potential (Fig. 1B and C). One reason for the decrease in OXPHOS complex levels is the increased oxidative stress caused by dysfunction mitochondria (Garcia-Ruiz et al. 2014; Ryan et al. 2021). This oxidative stress inhibits the synthesis and promotes the degradation of OXPHOS subunits. Also, as shown in Fig. 3C, IDH2 deficiency increased nitric oxide, regardless of co-culture with cancer cells. Considering that OXPHOS can be suppressed by increased NO in macrophages during M1 polarization (Palmieri et al. 2020; Sun et al. 2022), it is difficult to clearly determine whether the decreased OXPHOS observed in this study was due to IDH2 deficiency or the resulting increased M1 polarization.

Same as previous studies, OCR and ATP production were decreased in IDH2-deficient macrophages (Lee et al. 2020). Also, the difference was more pronounced in condition with cancer co-culture, the reason for the increased difference may be M2-like polarization is stronger in WT macrophage compared to IDH2-deficient macrophage. Based on previous research indicating that M1 macrophages exhibit high levels of ECAR, we anticipated that ECAR would be higher in IDH2-deficient macrophages due to their strong M1 characteristics (Van den Bossche et al. 2016; Dahlem et al. 2020). However, ECAR was measured slightly higher in the WT macrophage or almost the same. Previous results showing that ECAR is increased in M2 as well as M1 macrophages compared to undifferentiated macrophages (Dahlem et al. 2020; Lundahl et al. 2022), this discrepancy may be due to the reduced M2-differentiated macrophages in IDH2-deficient macrophages (Figs. 1E and 3E).A previous study has shown that mitochondrial fission plays an essential role in the secretion of proinflammatory cytokines (Park et al. 2013). Moreover, mitochondrial dynamics due to glutamine uptake are noteworthy, especially glutamine, considering that it is an important nutrient in the mitochondrial fission process (Rambold et al. 2011; Cai et al. 2018). From the results in Figs. 1 and S3, we predicted high levels of glutamine uptake and metabolism in IDH2-deficient macrophages and increased mitochondrial fission. Proteins involved in mitochondrial fusion include Mfn1, Mfn2, and OPA1, whereas fission includes Drp1. When Drp1 is recruited to the outer membrane, phosphorylation of its serine 616 residue induces mitochondrial fragmentation (Youle and van der Bliek 2012). The morphology of mitochondria differed between M1 and M2 macrophages, whereby M1 macrophages exhibited fragmented mitochondria due to mitochondrial dysfunction and activated Drp1. Conversely, M2 macrophages showed elongated mitochondria due to high OXPHOS levels and an upregulated expression of mitochondrial fusion-related proteins (Mishra and Chan 2016; Yao et al. 2019). As indicated in Fig. 2A, co-culture with cancer cells showed a tendency for increased mitochondrial fusion proteins and decreased fission proteins in macrophages. However, IDH2-deficient macrophages showed increased levels of p-Drp1(S616) and decreased levels of fusion proteins compared to WT. In addition, as shown in Fig. 2B and C, compared with WT, *IDH2*-/- macrophages possessed fragmented mitochondria. Notably, the percentage of fragmented mitochondria was increased in IDH2-deficient macrophages, whereas the percentage of elongated mitochondria decreased. Furthermore, mitochondria in IDH2-deficient macrophages were shorter than those in WT macrophages (Fig. 2D). In this study, IDH2 deficiency caused morphological changes in macrophage mitochondria. Therefore, these results demonstrated that shortened mitochondria by fission in IDH2-deficient macrophages were predominantly the M1 type.

Our study showed that IDH2-deficient macrophages exhibit the M1 phenotype, express increased levels of proinflammatory cytokines, and show elevated iNOS expression (Fig. 3A). In addition, the expression of genes encoding M1-related markers, *IL-1β*, and *IL-6*, was significantly increased, whereas those encoding M2-related markers, *Fizz1*, *YM1*, *IL-4*, *MGL1*, and *MGL2*, were significantly decreased as evident by the reduction in α-ketoglutarate production due to IDH2 deficiency (Fig. 3B) and enriched levels of NO (Fig. 3C). IDH2-deficient macrophages showed reduced capability of undergoing M2 polarization under IL-4 treatment (Fig. S2). The level of CD206, an M2 macrophage marker, decreased in IDH2-deficient macrophages, whereas iNOS and CD86 levels, M1 macrophage markers, tended to increase (Fig. 3D and E). Overall, these results demonstrate that IDH2-deficient macrophages exhibit higher M1 characteristics than M2.

Cancer cells invade surrounding tissues and blood vessels in the early stages of metastasis to form tumor masses. Epithelial cells are connected by a cell surface protein termed E-cadherin, and cell adhesion is established via tight and gap junctions. To be able to move, these epithelial cells should break off strong cell connections unique to epithelial cells and move as free cells. Therefore, EMT is a major characteristic that cancer cells must acquire for metastasis to occur (Brabletz et al. 2018). IL-6, a known M2 phenotype marker, induces EMT (Xu et al. 2014; Che et al. 2017). In cancer cells (LLC1) co-cultured with IDH2-deficient macrophages exhibiting the M1 phenotype, the levels of metastasis-related EMT marker fibronectin were decreased, whereas those of E-cadherin were increased (Fig. 4A). As shown in Fig. 4B, C and D, the proliferation and migration of cancer cells were significantly diminished by IDH2 deficiency in macrophages. These results speculated that IDH2 deficiency in macrophages suppresses cancer progression. In this study, using a tumor transplantation mouse model, we found that the average tumor size in the IDH2-deficient mouse group was significantly smaller than in the WT (Fig. 5A, B, D, and E). Furthermore, as shown in Fig. 4A, the tumor mass of the IDH2-deficient mouse group increased E-cadherin, whereas it decreased fibronectin. These results indicate that IDH2-deficient M1 macrophages in the tumor microenvironment inhibit cancer cell growth and suppress metastasis-related EMT in cancer cells owing to the inability of macrophages to undergo M2 polarization.

Macrophages secrete different cytokines in the microenvironment depending on their activation status and function, and these cytokines, in turn, determine the M1 or M2 subtype (Wang et al. 2014). Representative markers of M1 macrophage polarization include iNOS, which is related to inflammation, whereas M2 macrophage markers include Arg1, p-AMPK, and p-STAT3 (Genard et al. 2017). AMPK is an evolutionary conserved serine/threonine kinase that regulates energy homeostasis and metabolic stress. AMPK acts as a potent modulator of inflammatory signaling pathways in macrophages. Stimulation of macrophages with anti-inflammatory cytokines (IL-10 and TGFβ) resulted in rapid phosphorylation and activation of AMPK, whereas proinflammatory stimulation of macrophages with LPS resulted in AMPK dephosphorylation and inactivation (Sag et al. 2008). Therefore, AMPK activation is a feature of M2 macrophages. Expectedly, the levels of M2 macrophage-related markers Arg1, p-AMPK, and p-STAT3 showed a tendency to decrease in IDH2-deficient macrophages co-cultured with cancer cells (Fig. 3A). In addition, M1 macrophage-associated cytokines were upregulated in IDH2-deficient macrophages, whereas M2-macrophage-associated anti-inflammatory cytokines were downregulated (Fig. 3B). This trend was also confirmed in MACS tumor-associated macrophages (TAMs) isolated from transplanted tumor tissue of IDH2-deficient mice (Fig. 6B and C). Furthermore, as shown in Fig. 6D, E, and F, CD206 (an M2 macrophage marker) and Ki67 (a proliferation marker) were reduced, whereas iNOS (an M1 macrophage marker) was increased in the TAMs of IDH2-deficient mice; these data are consistent with our in vitro experimental results (Figs. 3D, E, and 4D). These in vitro and in vivo tumor transplantation model findings demonstrated that IDH2 deficiency predominantly increases M1 and inhibits M2 macrophage polarization, suppressing cancer progression.

Our study demonstrates that IDH2-deficient macrophages predominantly exhibit the M1 phenotype, with minimal induction of EMT and tumor growth, as evidenced by the in vitro and in vivo tumor models. Thus, we propose that IDH2 plays an important role in determining the fate of macrophage polarization and influences cancer progression. This study underlines the potential contribution of IDH2 deficiency in macrophages and its significance in the tumor microenvironment. These results would be useful for future advanced clinical cancer research.

## Supplementary Information

Additional file 1.

Additional file 2.

## Data Availability

All data generated or analyzed during this study are included in this published article.

## References

[CR1] Alkasalias T, Moyano-Galceran L, Arsenian-Henriksson M, Lehti K. Fibroblasts in the Tumor Microenvironment: Shield or Spear? Int J Mol Sci. 2018. 10.3390/ijms19051532. 29883428 10.3390/ijms19051532 PMC5983719

[CR2] Angajala A, Lim S, Phillips JB, Kim JH, Yates C, You Z, et al. Diverse roles of mitochondria in immune responses: novel insights into immuno-metabolism. Front Immunol. 2018;9:1605. 10.3389/fimmu.2018.01605. 30050539 10.3389/fimmu.2018.01605 PMC6052888

[CR3] Boutilier AJ, Elsawa SF. Macrophage polarization states in the tumor microenvironment. Int J Mol Sci. 2021. 10.3390/ijms22136995. 34209703 10.3390/ijms22136995 PMC8268869

[CR4] Brabletz T, Kalluri R, Nieto MA, Weinberg RA. EMT in cancer. Nat Rev Cancer. 2018;18(2):128–34. 10.1038/nrc.2017.118. 29326430 10.1038/nrc.2017.118

[CR5] Bruchard M, Mignot G, Derangere V, Chalmin F, Chevriaux A, Vegran F, et al. Chemotherapy-triggered cathepsin B release in myeloid-derived suppressor cells activates the Nlrp3 inflammasome and promotes tumor growth. Nat Med. 2013;19(1):57–64. 10.1038/nm.2999. 23202296 10.1038/nm.2999

[CR6] Cai WF, Zhang C, Wu YQ, Zhuang G, Ye Z, Zhang CS, et al. Glutaminase GLS1 senses glutamine availability in a non-enzymatic manner triggering mitochondrial fusion. Cell Res. 2018;28(8):865–7. 10.1038/s41422-018-0057-z. 29934617 10.1038/s41422-018-0057-z PMC6082853

[CR7] Che D, Zhang S, Jing Z, Shang L, Jin S, Liu F, et al. Macrophages induce EMT to promote invasion of lung cancer cells through the IL-6-mediated COX-2/PGE2/beta-catenin signalling pathway. Mol Immunol. 2017;90:197–210. 10.1016/j.molimm.2017.06.018. 28837884 10.1016/j.molimm.2017.06.018

[CR8] Dahlem C, Siow WX, Lopatniuk M, Tse WKF, Kessler SM, Kirsch SH, et al. Thioholgamide A, a new anti-proliferative anti-tumor agent, modulates macrophage polarization and metabolism. Cancers (Basel). 2020. 10.3390/cancers12051288. 32438733 10.3390/cancers12051288 PMC7281193

[CR9] Garcia-Ruiz I, Solis-Munoz P, Fernandez-Moreira D, Grau M, Colina F, Munoz-Yague T, et al. High-fat diet decreases activity of the oxidative phosphorylation complexes and causes nonalcoholic steatohepatitis in mice. Dis Model Mech. 2014;7(11):1287–96. 10.1242/dmm.016766. 25261569 10.1242/dmm.016766 PMC4213732

[CR10] Genard G, Lucas S, Michiels C. Reprogramming of tumor-associated macrophages with anticancer therapies: radiotherapy versus chemo- and immunotherapies. Front Immunol. 2017;8:828. 10.3389/fimmu.2017.00828. 28769933 10.3389/fimmu.2017.00828 PMC5509958

[CR11] Goel HL, Mercurio AM. VEGF targets the tumour cell. Nat Rev Cancer. 2013;13(12):871–82. 10.1038/nrc3627. 24263190 10.1038/nrc3627 PMC4011842

[CR12] Gordon S, Martinez FO. Alternative activation of macrophages: mechanism and functions. Immunity. 2010;32(5):593–604. 10.1016/j.immuni.2010.05.007. 20510870 10.1016/j.immuni.2010.05.007

[CR13] Ham IH, Lee D, Hur H. Role of cancer-associated fibroblast in gastric cancer progression and resistance to treatments. J Oncol. 2019;2019:6270784. 10.1155/2019/6270784. 31281359 10.1155/2019/6270784 PMC6590541

[CR14] Hao NB, Lu MH, Fan YH, Cao YL, Zhang ZR, Yang SM. Macrophages in tumor microenvironments and the progression of tumors. Clin Dev Immunol. 2012;2012:948098. 10.1155/2012/948098. 22778768 10.1155/2012/948098 PMC3385963

[CR15] Jha AK, Huang SC, Sergushichev A, Lampropoulou V, Ivanova Y, Loginicheva E, et al. Network integration of parallel metabolic and transcriptional data reveals metabolic modules that regulate macrophage polarization. Immunity. 2015;42(3):419–30. 10.1016/j.immuni.2015.02.005. 25786174 10.1016/j.immuni.2015.02.005

[CR16] Kim S, Kim SY, Ku HJ, Jeon YH, Lee HW, Lee J, et al. Suppression of tumorigenesis in mitochondrial NADP(+)-dependent isocitrate dehydrogenase knock-out mice. Biochim Biophys Acta. 2014;1842(2):135–43. 10.1016/j.bbadis.2013.11.008. 24240089 10.1016/j.bbadis.2013.11.008

[CR17] Laoui D, Van Overmeire E, De Baetselier P, Van Ginderachter JA, Raes G. Functional relationship between tumor-associated macrophages and macrophage colony-stimulating factor as contributors to cancer progression. Front Immunol. 2014;5:489. 10.3389/fimmu.2014.00489. 25339957 10.3389/fimmu.2014.00489 PMC4188035

[CR18] Lee JH, Go Y, Kim DY, Lee SH, Kim OH, Jeon YH, et al. Isocitrate dehydrogenase 2 protects mice from high-fat diet-induced metabolic stress by limiting oxidative damage to the mitochondria from brown adipose tissue. Exp Mol Med. 2020;52(2):238–52. 10.1038/s12276-020-0379-z. 32015410 10.1038/s12276-020-0379-z PMC7062825

[CR19] Li J, He Y, Tan Z, Lu J, Li L, Song X, et al. Wild-type IDH2 promotes the Warburg effect and tumor growth through HIF1alpha in lung cancer. Theranostics. 2018;8(15):4050–61. 10.7150/thno.21524. 30128035 10.7150/thno.21524 PMC6096397

[CR20] Li Y, He Y, Miao K, Zheng Y, Deng C, Liu TM. Imaging of macrophage mitochondria dynamics in vivo reveals cellular activation phenotype for diagnosis. Theranostics. 2020;10(7):2897–917. 10.7150/thno.40495. 32194843 10.7150/thno.40495 PMC7053213

[CR21] Liu PS, Wang H, Li X, Chao T, Teav T, Christen S, et al. alpha-ketoglutarate orchestrates macrophage activation through metabolic and epigenetic reprogramming. Nat Immunol. 2017;18(9):985–94. 10.1038/ni.3796. 28714978 10.1038/ni.3796

[CR22] Liu S, Yang J, Wu Z. The regulatory role of alpha-ketoglutarate metabolism in macrophages. Mediators Inflamm. 2021;2021:5577577. 10.1155/2021/5577577. 33859536 10.1155/2021/5577577 PMC8024083

[CR23] Lundahl MLE, Mitermite M, Ryan DG, Case S, Williams NC, Yang M, et al. Macrophage innate training induced by IL-4 and IL-13 activation enhances OXPHOS driven anti-mycobacterial responses. Elife. 2022. 10.7554/eLife.74690. 36173104 10.7554/eLife.74690 PMC9555863

[CR24] Margolin DA, Silinsky J, Grimes C, Spencer N, Aycock M, Green H, et al. Lymph node stromal cells enhance drug-resistant colon cancer cell tumor formation through SDF-1alpha/CXCR4 paracrine signaling. Neoplasia. 2011;13(9):874–86. 10.1593/neo.11324. 21969820 10.1593/neo.11324 PMC3182279

[CR25] Mishra P, Chan DC. Metabolic regulation of mitochondrial dynamics. J Cell Biol. 2016;212(4):379–87. 10.1083/jcb.201511036. 26858267 10.1083/jcb.201511036 PMC4754720

[CR26] Nunnari J, Suomalainen A. Mitochondria: in sickness and in health. Cell. 2012;148(6):1145–59. 10.1016/j.cell.2012.02.035. 22424226 10.1016/j.cell.2012.02.035 PMC5381524

[CR27] O’Neill LA. A broken krebs cycle in macrophages. Immunity. 2015;42(3):393–4. 10.1016/j.immuni.2015.02.017. 25786167 10.1016/j.immuni.2015.02.017

[CR28] Palmieri EM, Gonzalez-Cotto M, Baseler WA, Davies LC, Ghesquiere B, Maio N, et al. Nitric oxide orchestrates metabolic rewiring in M1 macrophages by targeting aconitase 2 and pyruvate dehydrogenase. Nat Commun. 2020;11(1):698. 10.1038/s41467-020-14433-7. 32019928 10.1038/s41467-020-14433-7 PMC7000728

[CR29] Park J, Choi H, Min JS, Park SJ, Kim JH, Park HJ, et al. Mitochondrial dynamics modulate the expression of pro-inflammatory mediators in microglial cells. J Neurochem. 2013;127(2):221–32. 10.1111/jnc.12361. 23815397 10.1111/jnc.12361

[CR30] Pollard JW. Tumour-educated macrophages promote tumour progression and metastasis. Nat Rev Cancer. 2004;4(1):71–8. 10.1038/nrc1256. 14708027 10.1038/nrc1256

[CR31] Rambold AS, Kostelecky B, Elia N, Lippincott-Schwartz J. Tubular network formation protects mitochondria from autophagosomal degradation during nutrient starvation. Proc Natl Acad Sci U S A. 2011;108(25):10190–5. 10.1073/pnas.1107402108. 21646527 10.1073/pnas.1107402108 PMC3121813

[CR32] Richards DM, Hettinger J, Feuerer M. Monocytes and macrophages in cancer: development and functions. Cancer Microenviron. 2013;6(2):179–91. 10.1007/s12307-012-0123-x. 23179263 10.1007/s12307-012-0123-x PMC3717063

[CR33] Ryan DG, Yang M, Prag HA, Blanco GR, Nikitopoulou E, Segarra-Mondejar M, et al. Disruption of the TCA cycle reveals an ATF4-dependent integration of redox and amino acid metabolism. Elife. 2021. 10.7554/eLife.72593. 34939929 10.7554/eLife.72593 PMC8735863

[CR34] Sag D, Carling D, Stout RD, Suttles J. Adenosine 5’-monophosphate-activated protein kinase promotes macrophage polarization to an anti-inflammatory functional phenotype. J Immunol. 2008;181(12):8633–41. 10.4049/jimmunol.181.12.8633. 19050283 10.4049/jimmunol.181.12.8633 PMC2756051

[CR35] Sica A, Mantovani A. Macrophage plasticity and polarization: in vivo veritas. J Clin Invest. 2012;122(3):787–95. 10.1172/JCI59643. 22378047 10.1172/JCI59643 PMC3287223

[CR36] Sun JX, Xu XH, Jin L. Effects of metabolism on macrophage polarization under different disease backgrounds. Front Immunol. 2022;13:880286. 10.3389/fimmu.2022.880286. 35911719 10.3389/fimmu.2022.880286 PMC9331907

[CR37] Tang JX, Thompson K, Taylor RW, Olahova M. Mitochondrial OXPHOS biogenesis: co-regulation of protein synthesis, import, and assembly pathways. Int J Mol Sci. 2020. 10.3390/ijms21113820. 32481479 10.3390/ijms21113820 PMC7312649

[CR38] Trouplin V, Boucherit N, Gorvel L, Conti F, Mottola G, Ghigo E. Bone marrow-derived macrophage production. J vis Exp. 2013. 10.3791/50966. 24300014 10.3791/50966 PMC3991821

[CR39] Van den Bossche J, Baardman J, Otto NA, van der Velden S, Neele AE, van den Berg SM, et al. Mitochondrial dysfunction prevents repolarization of inflammatory macrophages. Cell Rep. 2016;17(3):684–96. 10.1016/j.celrep.2016.09.008. 27732846 10.1016/j.celrep.2016.09.008

[CR40] Wang N, Liang H, Zen K. Molecular mechanisms that influence the macrophage m1–m2 polarization balance. Front Immunol. 2014;5:614. 10.3389/fimmu.2014.00614. 25506346 10.3389/fimmu.2014.00614 PMC4246889

[CR41] Weischenfeldt J, Porse B. Bone marrow-derived macrophages (BMM): isolation and applications. CSH Protoc. 2008. 10.1101/pdb.prot5080. 21356739 10.1101/pdb.prot5080

[CR42] Williams NC, O’Neill LAJ. A role for the krebs cycle intermediate citrate in metabolic reprogramming in innate immunity and inflammation. Front Immunol. 2018;9:141. 10.3389/fimmu.2018.00141. 29459863 10.3389/fimmu.2018.00141 PMC5807345

[CR43] Winkler J, Abisoye-Ogunniyan A, Metcalf KJ, Werb Z. Concepts of extracellular matrix remodelling in tumour progression and metastasis. Nat Commun. 2020;11(1):5120. 10.1038/s41467-020-18794-x. 33037194 10.1038/s41467-020-18794-x PMC7547708

[CR44] Xu H, Lai W, Zhang Y, Liu L, Luo X, Zeng Y, et al. Tumor-associated macrophage-derived IL-6 and IL-8 enhance invasive activity of LoVo cells induced by PRL-3 in a KCNN4 channel-dependent manner. BMC Cancer. 2014;14:330. 10.1186/1471-2407-14-330. 24885636 10.1186/1471-2407-14-330 PMC4024187

[CR45] Yao CH, Wang R, Wang Y, Kung CP, Weber JD, Patti GJ. Mitochondrial fusion supports increased oxidative phosphorylation during cell proliferation. Elife. 2019. 10.7554/eLife.41351. 30694178 10.7554/eLife.41351 PMC6351101

[CR46] Yeung KT, Yang J. Epithelial-mesenchymal transition in tumor metastasis. Mol Oncol. 2017;11(1):28–39. 10.1002/1878-0261.12017. 28085222 10.1002/1878-0261.12017 PMC5242415

[CR47] Yi L, Gai Y, Chen Z, Tian K, Liu P, Liang H, et al. Macrophage colony-stimulating factor and its role in the tumor microenvironment: novel therapeutic avenues and mechanistic insights. Front Oncol. 2024;14:1358750. 10.3389/fonc.2024.1358750. 38646440 10.3389/fonc.2024.1358750 PMC11027505

[CR48] Yoo HC, Yu YC, Sung Y, Han JM. Glutamine reliance in cell metabolism. Exp Mol Med. 2020;52(9):1496–516. 10.1038/s12276-020-00504-8. 32943735 10.1038/s12276-020-00504-8 PMC8080614

[CR49] Youle RJ, van der Bliek AM. Mitochondrial fission, fusion, and stress. Science. 2012;337(6098):1062–5. 10.1126/science.1219855. 22936770 10.1126/science.1219855 PMC4762028

[CR50] Yuan Y, Jiang YC, Sun CK, Chen QM. Role of the tumor microenvironment in tumor progression and the clinical applications (Review). Oncol Rep. 2016;35(5):2499–515. 10.3892/or.2016.4660. 26986034 10.3892/or.2016.4660

[CR51] Zhang X, Goncalves R, Mosser DM. The isolation and characterization of murine macrophages. Curr Protoc Immunol Chapter. 2008. 10.1002/0471142735.im1401s83. 10.1002/0471142735.im1401s83 PMC2834554 19016445

[CR52] Zorova LD, Popkov VA, Plotnikov EY, Silachev DN, Pevzner IB, Jankauskas SS, et al. Mitochondrial membrane potential. Anal Biochem. 2018;552:50–9. 10.1016/j.ab.2017.07.009. 28711444 10.1016/j.ab.2017.07.009 PMC5792320

