# Supplementary material for: IDH2 regulates macrophage polarization and tumorigenesis by modulating mitochondrial metabolism in macrophages
Source: Mol Med. 2024 Sep 10;30:143. doi: 10.1186/s10020-024-00911-x (PMC11385829; doi:10.1186/s10020-024-00911-x)
Supplement: Supplementary file 1 — Additional file 1. [file 10020_2024_911_MOESM1_ESM.docx]

**Supplementary Materials**


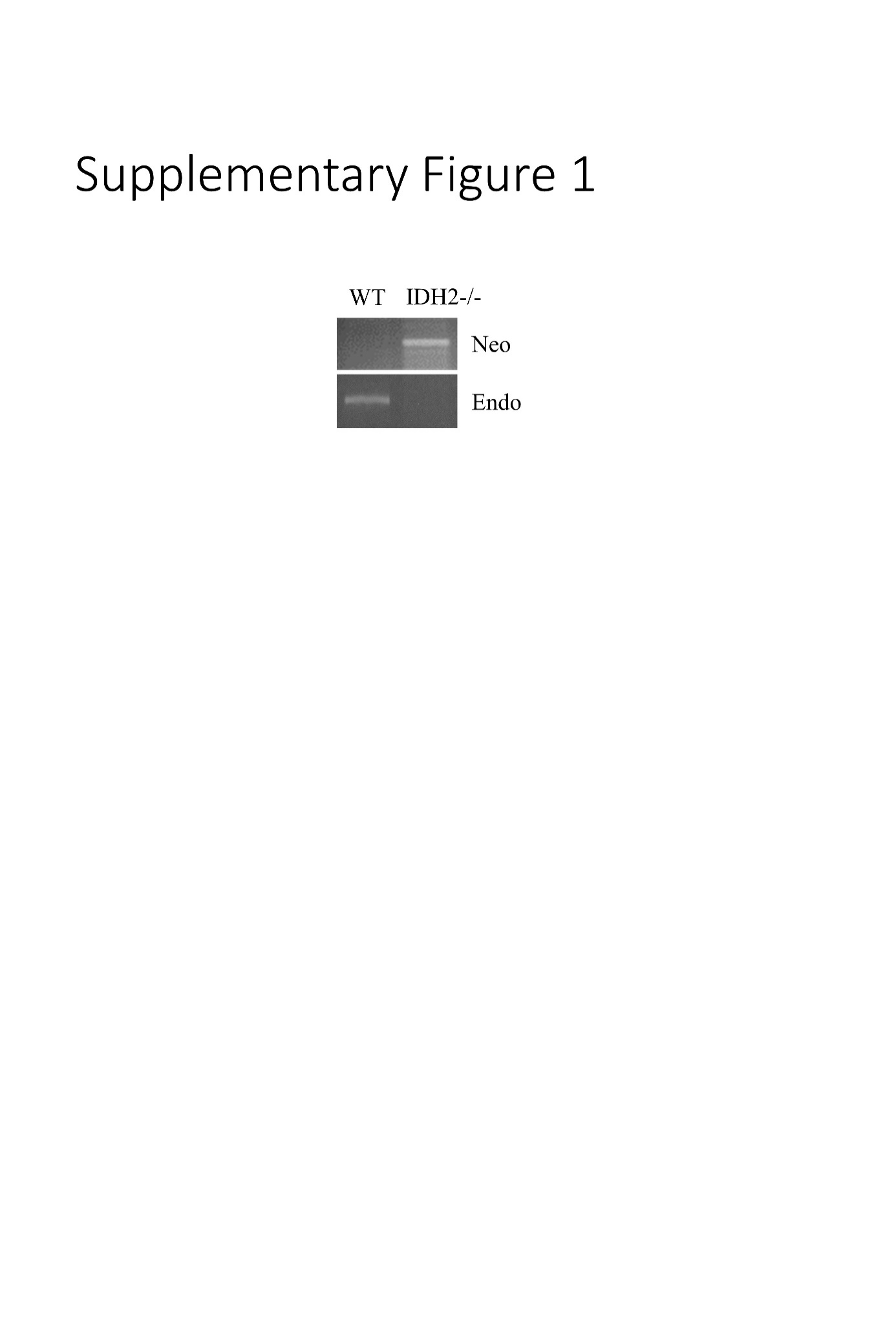


**Fig. S1** IDH2 Knockout mice confirmation.

PCR analysis of IDH2 gene expression in WT and idh2^−/−^ mice genomic DNA.


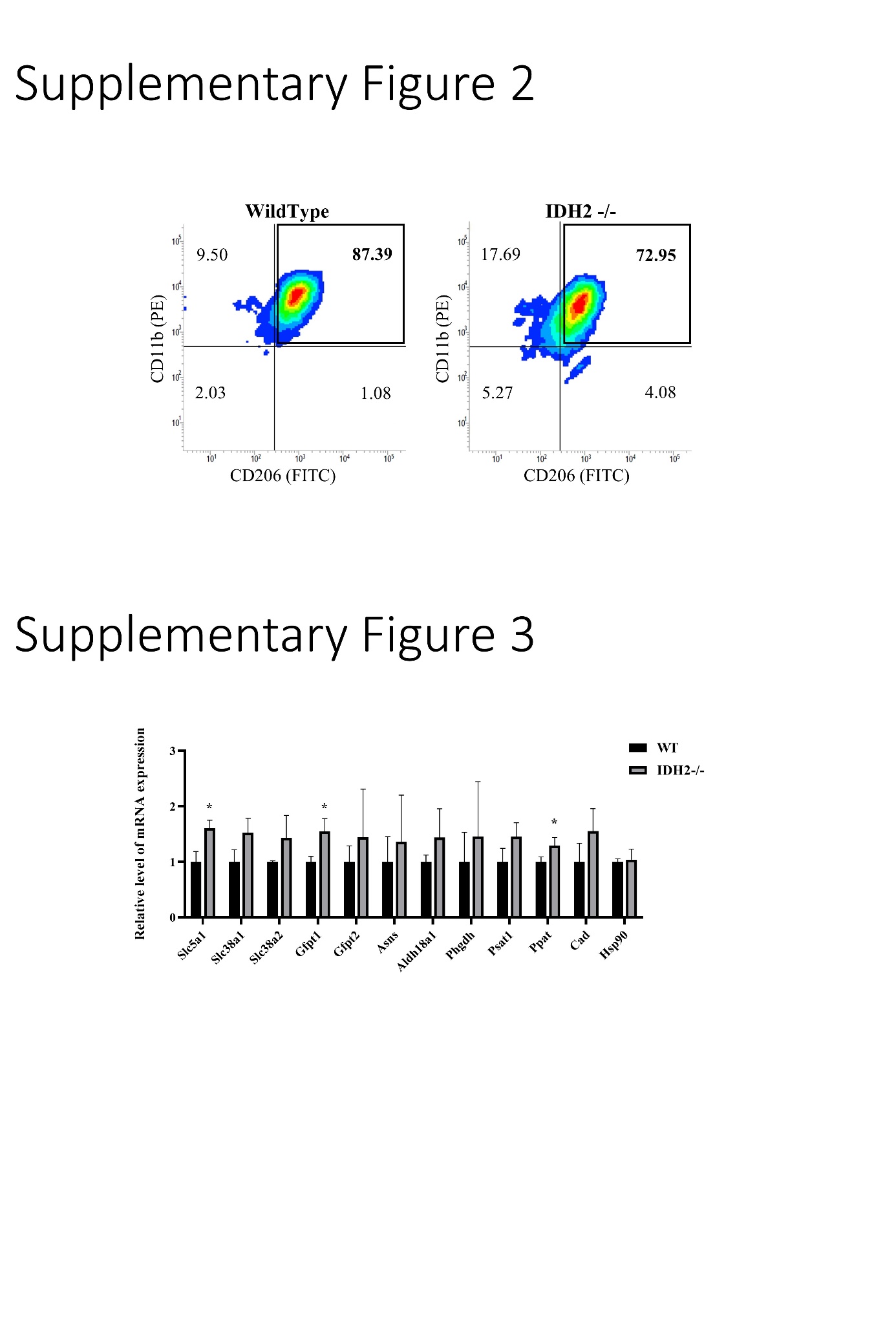


**Fig. S2** Comparison of BMDMs polarization to M2 as a percentage of CD11b+/ CD206+ using flow cytometry. WT and IDH2-/- BMDMs were treated IL-4 for 24hr.


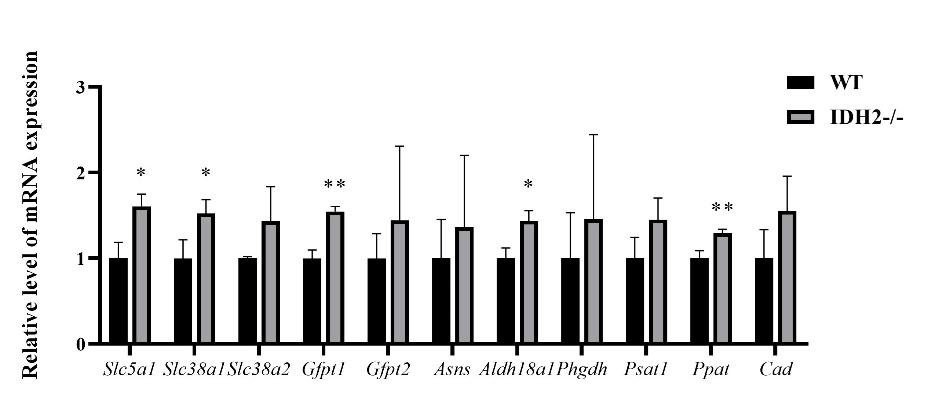


**Fig. S3** Relative level of mRNA expression of glutamine influx signatures and rate-limiting enzyme genes for each glutamine metabolism (*Slc5a1, Slc38a1and Slc38a2*; glutamine influx, *Gfpt1* and *Gfpt2*; UDP-GlcNAc synthesis*, Asns*; asparagine synthesis*, Aldh18a1*; proline synthesis*, Phgdh* and *Past1*; serine synthesis*, Ppat*; purine synthesis*, Cad*; pyrimidine synthesis). Data are presented as the mean ± SEM of at least three independent experiments (* p < 0.05, ** p < 0.01, *** p < 0.001, *t*-test).
